# Supplementary material for: Are carbon emissions trading and green financial instruments synergistic? -Comprehensive quantitative research based on content analysis
Source: PLoS One. 2024 Mar 7;19(3):e0298601. doi: 10.1371/journal.pone.0298601 (PMC10919601; doi:10.1371/journal.pone.0298601)
Supplement: S1 Data — (ZIP) [file pone.0298601.s001.zip › Data/地级市Z_F工作报告.zip!/╒π╜¡/╜≡╗¬╩╨/└┤╫╘╣1⁄2╓┌║┼í╛┬φ┐╦╩2╛▌═°í┐_2015.docx]

各位代表：

现在，我代表金华市人民政府向大会报告工作，请予审议，并请市政协委员和其他列席人员提出意见。

　　一、2014年主要工作回顾

　　过去的一年，是全面深化改革的开局之年，是“五水共治”倒逼转型的攻坚之年，也是新常态下负重拼搏的赶超之年。市政府全面贯彻省委、省政府和市委的各项决策部署，紧紧依靠全市人民，迎难而上，奋发有为，较好地完成了市六届人大五次会议确定的目标任务。全市实现地区生产总值3207亿元，增长8.3%；一般公共预算收入268.9亿元，增长10.9%；城镇、农村常住居民人均可支配收入39807元、18544元，分别增长9.4%和11.3%；以上增幅均居全省前列，保持较好的赶超势头。

　（一）经济发展稳中向好。面对经济下行压力，全力打好转型升级“组合拳”，经济发展质量和效益稳步提升。现代农业加快发展。粮食及蔬菜、茶叶、花卉苗木等主要农产品生产稳中有增。新建粮食生产功能区11.9万亩，新增省级现代农业综合园区3个、设施农业1万亩。新增年销售额超亿元农业龙头企业3家、省级示范性家庭农场21家。推广种养结合生态循环模式，畜牧业转型升级步伐加快。农产品质量省级抽检合格率98.6%。东阳获全国粮食生产先进单位。

工业经济提质增效。规上工业增加值945亿元、增长7.4%，单位工业增加值能耗下降9.5%。大力推进“四换三名”和“两化”深度融合，年产值超百亿元园区8个、超百亿元行业17个，其中装备制造业突破1700亿元，战略性新兴产业、高新技术产业增加值分别增长6.1%和8.1%。加快培育大企业大集团和高成长企业，新增上市及新三板、浙江股权交易中心挂牌企业11家，入选中国民营企业500强9家；新增中国驰名商标5件、省著名商标38件、浙江名牌产品36只，企业参与制订国家标准8个、行业标准21个。华铝完成战略重组，吉利发动机等重大项目开工建设。科技创新带动作用进一步增强，新增国家级高新技术企业65家、省级高新技术研发中心32家、省级重点企业研究院6家，规上工业新产品产值增长25.7%。加大招才引智力度，入围国家“****”7人、入选省“****”15人。建筑业总产值突破3000亿元，获评省级建筑强市。房地产业平稳发展。

服务业快速发展。第三产业增加值1559亿元、增长9.5%，占地区生产总值的 48.6%，比重提高0.9个百分点。信息网络经济蓬勃发展，建成网络经济园区24个，北大信息科技园开园，浙师大网络经济创业园、电信云计算等重大项目开工建设，新增网络企业1.5万家，实现电子商务交易额2356亿元、软件和信息服务业营业收入330亿元，分别增长29.7%和36%。旅游业发展形势喜人，接待游客和旅游收入分别增长21.7%和26%，过夜游比重稳步攀升，新增武义牛头山等国家4A景区4个、省级旅游度假区1个，兰溪成为全国休闲农业与乡村旅游示范县。文化产业加快发展，入选省级文化产业示范基地（园区）7家，国家级文化出口重点企业5家、省级13家，东阳举办世界工艺文化节和中国木雕博览会。

三大需求拉动有力。完成固定资产投资1595亿元，增长16.9%，省“411”重大项目及省、市重点项目超额完成年度投资计划。进出口总额首次突破400亿美元，跻身“全国外贸百强城市”第24位。社会消费品零售总额1593亿元、网络零售额942亿元，分别增长13.2%和41%。浙（婺）商回归到位资金超300亿元，实际利用外资2.8亿美元，引进投资超10亿元项目14个。完善财政金融政策，支持实体经济发展，新增贷款576亿元，贷款余额增长11.2%。积极防范和化解企业资金链担保链风险，政府应急周转金累计转贷248亿元。

（二）重点改革取得突破。“四张清单一张网”初步建成，市级部门行政权力事项精减54%，财政专项资金整合为37项，制定实施部门责任清单，开展负面清单外企业投资项目不再审批试点，开通政务服务网。行政审批制度改革进一步深化，下放市级审批权限204项，开设“中介服务超市”，完善全程代理和集中审批机制。商事制度改革成效明显，推行先照后证、多证联办、证照合办，工商注册实现内资2天、外资3天、简易事项当天办结，新增市场主体11万户、注册资本576亿元，分别增长8%和40.5%。组建市场监管局、卫生计生委，健全基层国土管理等体制。

义乌国际贸易综合改革试点取得重大突破，市场采购贸易方式全面落地，保税物流中心封关运营，航空口岸对外开放，“义新欧”中欧班列开通运行。国家现代服务业综合试点启动实施，首批82个项目完成投资54亿元。商务部四大体系建设试点不断深化。积极参与长江经济带海关区域通关一体化改革，跨境贸易电子商务服务试点成效突出，金义“跨境通”平台日通关量突破4.5万票。低丘缓坡综合开发利用试点取得实效，完成农转用报批和耕地占补平衡，平整土地2.7万亩，供地项目87个。义乌国家新型城镇化和农村改革试验区试点落地。

开展“四破”专项整治，建立健全工业企业亩产效益综合评价、土地差别化管理、低效用地再开发等机制，消化批而未供土地2.4万亩，盘活存量用地1.1万亩。推行企业排污权有偿使用和交易机制。加快推进农村“三权”改革，完成农村土地承包经营权调查，实施1297个村集体经济股份合作制改革，农村宅基地发证登记率74%。扩大林地股份流转和林权抵押贷款试点。武义启动户籍管理制度改革试点。永康创新农村金融自治模式。

　（三）环境治理成效明显。以全省“五水共治”现场会为契机，全面打响“保护母亲河”攻坚战，迅速掀起全民治水、全域治水、全力治水的热潮。全面实施十大行动，在全省率先建立挂图作战指挥体系、率先形成河长河道警长全覆盖网络、率先建立跨流域治水联动机制，坚持水岸同治、上下齐治、标本兼治，基本消灭垃圾河，婺城长湖等83条、749公里黑臭河得到重点治理， 4个劣Ⅴ类水质断面全部消除，所有县市交接断面水质达到Ⅳ类以上，兑现了“一年灭黑臭”的承诺，可游泳的河越来越多，广大群众由衷点赞。一大批治水重点项目开工建设，新增污水处理能力15.8万吨，城市污水集中处理率91%，提高2个百分点。加固病险水库27座、堤防130公里，整治山塘159座、河道543公里。市第二水厂开工建设。严格管理地下水资源，积极创建节水型城市。加大保护“大水缸”力度，水源涵养生态功能区扩大292平方公里。我市捧回了全省“五水共治”优秀市大禹鼎，东阳、浦江捧回了优秀县大禹鼎，婺城获全国水利先进集体，兰溪获全省水利“大禹杯”金奖。

“三改一拆” 形成破竹之势。在全省率先拆除高速公路沿线违法广告牌，全面开展农村非法“一户多宅”、宗教违建场所、违法临时建筑等专项整治，拆除违法建筑1819万平方米，创建无违建乡镇（街道）70个、无违建村（社区）1947个。浦江通过省“无违建县”创建验收。有力推进婺城二七区块、石门农场棚户区改造，加大背街小巷改善力度，完善撤村建居政策体系，城中村改造推行公寓式安置、社区化建设。改造城中村821万平方米、旧住宅区421万平方米、旧厂区370万平方米。

向大气污染宣战。实施治气三年行动计划，开展管车、降尘、控煤、禁燃等十大专项行动，淘汰黄标车和老旧车6万余辆，强化建筑工地、道路扬尘治理，市区PM2.5浓度下降8.6%，空气质量优良天数达到235天，比上年增加30天。

推进城市治堵。实施城市主干道改造提升工程，打通断头路12条，市区一环路、二环路贯通。优先发展公交，开工建设首条BRT（快速公交）线路，新增公共自行车1.2万辆，公交分担率提高到18.5%。加大地下停车场归位整治力度，交通陋习、超限超载等整治取得新进展。

加大农村“三治”力度。开展1802个村生活污水治理，受益农户25.6万户。在75个乡镇1345个村实施农村生活垃圾分类化、资源化、减量化处理。兰溪实行农村卫生收费制度。整治赤膊房172万平方米。建成美丽乡村风景线12条、美丽乡镇14个、精品村60个、秀美村140个，新增省级美丽宜居示范村8个。永康获评全省美丽乡村创建先进县。

（四）都市区建设加速推进。规划共绘工作取得新进展。完成金华-义乌都市区规划纲要、金义东城际轨道交通线位用地控制规划，编制浙中城市群重大基础设施空间一体化规划，完成市区24个专项规划和重点区块控规设计，村庄建设规划覆盖率98.6%。

中心城区建设进一步加强。加快多湖中央商务区建设，完成概念规划编制和核心区块城市设计，万达广场建成开业，一批总部经济项目落户，横塘沿安置小区一期结顶、二期开工，丹溪大桥通车，东市街南延、宾虹路东延等工程进展顺利。湖海塘公园、高山头古城旅游开发项目开工建设，古子城区块开展综合整治，尖峰山区块基础设施加快推进。五百滩公园、燕尾洲公园及八咏桥投入使用，三江六岸成为一道靓丽的风景线。金义都市新区管理体制进一步完善，核心区块环路贯通，满塘湖景观改造一期完工，地下综合管廊、新社区集聚建设工程加快推进，“菜鸟”项目一期建成运营，中国移动·金义信息通信产业园等重大项目开工建设，电商产业园集聚企业120多家，网络经济学院开学。婺城新城区、金东新城区、金华开发区金西区块建设进展顺利。义乌启动丝路新区、陆港新区、科创新区规划建设。加强“信义金华”城市形象宣传。开展乱搭乱建、市场环境秩序等专项整治。市区启动全国文明城市创建，成为国家园林城市，武义、东阳成为省级森林城市，磐安成为省级示范文明县城。东阳开展省级首批美丽县城建设试点。新增14个国家重点镇、9个市级中心镇，永康古山镇列入省级小城市培育试点。

城乡基础设施建设加快推进。杭长高铁开通，全面融入上海两小时交通圈，金华发展跨入“高铁时代”。金温铁路扩能改造项目完成拆迁，金台铁路项目建议书获批。东永高速完成主体工程，杭金衢高速改扩建工程动工，330国道改建、20省道浦江段、永磐公路永康段、42省道磐安段、义兰公路等工程加快推进。开通金兰中线城际直达公交。开工建设衢江航运姚家、游埠枢纽工程。建成两项国家特高压工程，新增110千伏及以上线路228公里。开展智慧城市建设试点，列入“宽带中国”示范城市。加强地下空间开发利用，新开发地下空间401万平方米。

（五）民生事业持续改善。全力办好惠民实事，民生支出占一般公共预算支出的75.8%，十个方面民生实事如期完成。就业社保体系不断完善。新增城镇就业7.6万人，城镇登记失业率2.8%。最低工资、养老、医疗、失业保险、被征地农民基本生活保障和城乡居民低保等标准逐年提高，城乡居民养老、医疗保险参保率分别达97%和98.8%，救助医疗困难群众3.2万人次。社会养老服务体系进一步健全，新增居家养老服务照料中心1553家、机构养老床位3222张，金东获评中国养老模式创新十佳典型。保障性安居房建设加快推进，新开工23390套，竣工5327套。改善农村饮水条件，惠及人口18.5万。做好扶贫工作，促进山区加快发展。

加快发展社会事业。投入2.8亿元改造薄弱学校29所，标准化中小学校比例达66%，婺城、金东、东阳、义乌、浦江、磐安通过全国义务教育发展基本均衡县验收，兰溪、永康、武义通过省级验收。全面实施公立医院综合改革，深化基层医疗卫生事业“四改联动”，基本形成“20分钟医疗服务圈”，公立医院药品收入占比下降4.7%，基层医疗机构业务量增长15%。“单独两孩”政策落地实施，出生人口性别比实现六连降。文化事业繁荣发展，文化惠民工程深入实施，新建成农村文化礼堂221家，举办“欢乐金华”百姓文化节，新增国家级非遗名录项目4个，浦江嵩溪等百个历史文化古村落保护利用取得新进展。武义延福寺修复工程启动。体育赛事和群众健身活动广泛开展。

创新社会治理体系。8890“市域一张网”实现市、县、乡三级联动，受理市民诉求12.9万件，办结率和回访满意率99%以上，基本建成应急类报警打110、非应急求助找8890、社会力量参与公共服务的社会治理新机制。健全社会治安防控体系、重大决策社会稳定风险评估机制，加强信访、调解和社区矫正工作，组建网络经济法律服务中心和矛盾纠纷调处中心，刑事发案量下降4.9%，平安金华建设向纵深推进。强化食品药品安全监管和基层责任网络建设，建成农产品安全快速检测室170个，放心农贸市场21家，主城区实现家禽定点屠宰杀白上市。完善安全生产责任体系，开展重点领域打非治违专项行动，强化涉爆粉尘行业整治，关闭涉爆金属粉尘企业101家、整改272家。

全面发展老龄、慈善、残疾人和关心下一代事业。支持工会、共青团、妇联等人民团体开展工作，加强民族、宗教、外侨、对台、气象、民防、统计、档案、地方志、仲裁、应急管理等工作。支持国防和军队建设，实现省级双拥模范城五连冠。深化援疆、援藏、援青及“双对口”工作。完成第三次经济普查。

过去一年，市政府按照中央和省委、市委的部署，深入开展以为民务实清廉为主题的群众路线教育实践活动，聚焦“四风”问题，开门听取意见，深入开展批评和自我批评，立说立行、带头抓好34项问题的整改落实。积极回应基层和群众诉求，开展农村（社区）机构牌子多、创建活动多、考核检查多等专项整治，破解了一批历史遗留问题。完成市级机关公车改革，规范公务接待标准，清理腾退办公用房2.2万平方米。“三公”经费支出下降38.6%。加大政务公开力度，推进预决算信息公开，审计监督进一步加强。认真落实人大决定决议，自觉接受人大法律监督和工作监督、政协民主监督和群众社会监督，建立代表委员列席市政府常务会议制度。扎实办理人大代表建议288件、政协提案426件。

各位代表，过去一年，我市取得的成绩来之不易，这是省委、省政府和市委正确领导的结果，是全市人民共同努力的结果。广大干部尤其是基层干部蛮拼的。在此，我代表市人民政府，向全市人民，向市人大代表、政协委员，向离退休老干部，向各民主党派、工商联、人民团体和社会各界人士，向驻金解放军和武警部队官兵，向中央和省驻金单位，向所有关心支持金华发展的朋友们，致以崇高的敬意和由衷的感谢！

同时，我们也清醒地认识到，经济社会发展和政府工作还存在不少问题，主要是：经济结构性矛盾仍然突出，创新能力不足，发展平台不优，投资后劲不强，产业转型升级处在阵痛期；资源环境压力加大，环境容量和节能降耗空间不足，治水治气治堵任务依然艰巨；城乡基础设施承载力不足，背街小巷、城乡结合部环境治理滞后；生产安全、食品安全还存在薄弱环节；政府职能转变还不到位，法治思维尚未完全确立，依法行政能力有待加强，重审批轻监管、重执法轻服务的现象仍然存在，“四风”问题存在反弹的风险。我们将正视问题，采取有力措施，切实加以改进。

　　二、2015年目标任务和重点工作

　　2015年是全面深化改革的关键之年，是全面推进法治建设的开局之年。做好今年工作既有许多有利条件，也面临不少困难挑战。当前，我国经济发展进入新常态，呈现增长速度变化、经济结构优化、发展动力转化的特征。我们要主动适应新常态，积极谋求新发展，着力在转型升级上下更大功夫，努力打造更有效率的政务生态系统、更有活力的创业创新生态系统、更有魅力的自然生态系统、更加和谐的社会生态系统。

今年政府工作的总体要求是：全面贯彻党的十八大和十八届三中、四中全会精神，认真落实省委、省政府和市委的决策部署，以“八八战略”为总纲，以提高发展质量和效益为中心，坚持稳中求进工作总基调，狠抓改革攻坚，强化创新驱动，突出环境治理，加快转型升级，统筹城乡发展，持续改善民生，全面深化法治金华建设，全面完成“十二五”目标任务，为建设“两富”“两美”现代化金华奠定更加坚实的基础。

　　建议2015年全市经济社会发展主要预期目标：地区生产总值增长8%左右，一般公共预算收入增长8.5%以上，城乡常住居民人均可支配收入增长9.5%以上，节能减排等约束性指标完成省定目标任务。

围绕以上目标要求，重点抓好八个方面的工作：

　（一）抓改革，不断增强发展活力。以改革统领经济社会发展全局，用政府权力的“减法”换取市场活力的“加法”。主动对接国家战略。积极融入“一带一路”、长江经济带国家战略，认真编制“十三五”经济社会发展规划，谋划好重大平台、重大项目、重大举措。全力推进“义新欧”中欧班列常态化运行，扩大与“一带一路”沿线节点城市的交流合作，发展公铁联运、海铁联运、江海联运，深化国际贸易合作。主动接轨上海自贸区，加强制度创新，承接先进制造业转移和智力输出。抢抓杭长高铁开通的契机，大力发展“高铁经济”。积极参与长江经济带海关区域通关一体化、丝绸之路经济带检验检疫一体化改革，加快“大通关”建设，积极申报金义综合保税区。加强金义“跨境通”平台建设，完善电子口岸功能，积极开展跨境电商进口试点，规划建设跨境电商园区。加快设立义乌国际邮件互换局和国际邮件交换站。加快推进国家改革试点。全面实施义乌国际贸易综合改革试点第二个三年计划，深化贸易、投融资、物流、通关、公共服务等便利化改革，创新监管制度，推动一般贸易、市场采购、跨境电商融合发展。加快推进国家现代服务业综合试点，创新投融资体制，加强特色平台和项目建设。认真抓好低丘缓坡综合开发利用试点。加快义乌国家新型城镇化和农村改革试验区试点。深入推进要素市场化配置改革。总结东阳、浦江等省级扩面试点做法，加大“四破”整治力度。完善亩产效益评价、差别化要素配置、低效用地退出机制，推行工业用地分阶段出让、全生命周期管理，提高亩均产出率。积极开展工矿废弃地复垦利用试点。深化投融资体制改革，推广政府与社会资本合作模式，完善利用社会资本建设大项目的体制机制，用好政府债券融资杠杆，引导社会资本投向基础设施和社会事业。落实生态环境财政奖惩制度，探索建立与污染物排放总量挂钩的水环境区域补偿机制。推进国资国企改革和整合重组。建立重大产业发展引导基金，探索建立新型金融组织。不断深化城乡体制改革。加快推进宅基地确权登记发证工作，实现土地承包经营权确权登记全覆盖，完善农村宅基地有偿使用、退出机制，加快实施村级集体经济股份制改革，建设农村综合产权交易平台。推进不动产统一登记工作。深化户籍管理制度改革。

　（二）稳增长，继续保持赶超态势。坚持量质并举，努力实现平稳健康发展。强化投资在稳增长中的关键作用。优化投资结构，抓好基础设施、发展平台、主导产业、技术改造、社会民生和生态环保等六大领域投资，实施工业技改投入三年倍增计划，推进城中村改造、“五水共治”双百亿投资计划。重视浙（婺）商回归、利用外资、对接央企工作，强化产业链招商、平台招商和环境招商，重点抓好一批重大项目的招商和合作。重视项目谋划储备，加强项目前期工作。强化出口在稳增长中的支撑作用。依托义乌国际贸易改革，带动出口持续增长。加大对出口基地、出口品牌、企业参展、外贸综合服务平台的支持力度，大力发展进口和加工贸易，促进进出口平衡发展。强化消费在稳增长中的基础作用。打造特色商业街区，办好浙中购物节，建设浙中商业购物中心，积极培育网购、旅游、健康、体育、文化等消费热点。强化实体经济在稳增长中的主体作用。加快实施大企业大集团和高成长企业培育计划，大力推进“个转企、小升规、规改股、股上市”，着力培育100家大企业大集团、100家高成长企业，新增股份公司50家以上，“个转企”1000家、“小升规”200家以上。实施小作坊清洁生产三年行动计划。加大小微企业扶持力度，创新落实企业减负政策，放水养鱼，提升小微企业生存能力和竞争能力。积极化解企业资金链担保链风险，着力提升企业抗风险能力。加强政银企合作，对诚信企业推行银行贷款年审制。综合施策，促进建筑业、房地产业平稳健康发展。深化财政体制改革，加强政府债务管理。

　（三）调结构，构建现代产业体系。对接全省七大万亿产业发展导向，按照“一个产业，一个规划，一套政策，一个班子”的工作要求，完善推进机制，健全服务体系，重点培育发展“五大千亿产业”。大力发展信息网络经济。重点培育电子商务、信息软件、通信服务、电商物流、互联网金融产业和云产业，加快菜鸟·金义电子商务新城、浙师大网络经济创业园、北大信息科技园等建设，大力引进国内外知名网络企业，强化上下游产业配套，推进网上网下市场融合，加快建设全国网商集聚中心、网络经济强市，力争网络零售额、软件信息服务业营业收入和电子信息制造业产值合计超2000亿元。大力发展先进装备制造产业。做大做强新能源汽车、汽车及零部件、新型农机装备、高档数控机床、智能电子技术设备等行业，加快推进新型建筑工业化，以信息化提升装备水平，改造工艺流程，促进生产智能化、制造业高端化，力争产值突破2000亿元。大力发展健康生物医药产业。加快建设金华现代生物医药产业园、武义有机国药产业养生园，培育康恩贝健康产业带，打造医药电商物流平台，努力引进知名医药企业和研发机构，支持新药研发和产业化，加快建设浙中特色医药产业基地，力争总产值超600亿元。大力发展文化影视时尚产业。培育壮大网络服务、影视创作、文化创意、工业设计、服装服饰、工艺书画等特色行业，加快横店影视文化产业实验区建设，办好义乌文交会、东阳木雕博览会、浦江书画节等展会，促进文化与产业融合发展，创建文化产业品牌，提升文化产业比重，增强文化软实力，力争营业收入超2000亿元。大力发展休闲旅游服务产业。合力打响五大主题旅游品牌，重视特色旅游产品开发，推进景区串点连线成片，谋划大金华山旅游开发。加快发展“美丽经济”，促进休闲旅游与传统村落文化的融合，培育乡村民宿、养生养老、运动休闲等产业，力争旅游人数和收入均增长16%以上。

（四）重创新，不断提升发展质效。强化创新驱动发展，推动大众创业万众创新。搭建好创新平台。按照“七位一体”的目标定位，规划建设金华国际科技城，加快启动中德科技园建设，做优做强中科院金华科技园、金华国家农业科技园区、永康现代农业装备高新区、武义科技城等。以工业强县（市）、工业强镇为重点，整合优化提升各类产业集聚区、开发区和工业园区，加快总部经济、信息经济和服务业集聚区建设，整体推进产业集聚、产业创新和产业升级。永康规上工业产值力争突破千亿元，支持义乌、兰溪、东阳等工业强市争先进位。加快建设大数据、云计算、云服务等信息网络技术平台，以及汽车零部件、五金门业、红木家具、棉纺织等质检中心，大力引进中小企业跨境电商云平台。培育好创新主体。实施“机器换人1168工程”，抓好“两化”融合示范企业、“三名”试点企业。支持企业研究院、高新技术研发中心建设，用好产业基金、创业投资引导基金，新认定国家级高新技术企业60家以上，实现R＆D（全社会研究与试验发展经费支出）占地区生产总值比重2%以上，发明专利授权630件以上。转化好创新成果。坚持“企业主体、市场主导、政府主推”，鼓励大院名校在金设立技术转移中心、中试基地、试验中心、数据中心。大力发展网上技术市场，加快院士专家工作站、博士后科研工作站、海外高层次人才创业创新基地建设。开展全国质量强市示范市创建工作。集聚好创新人才。深入实施“****”、“双龙计划”，加强海外人才及外国专家对接与项目合作，成立人才银行。引进高端人才1000人、紧缺急需人才1万人以上，新培养高技能人才2万人以上，争创信息网络、文化影视领域人才管理改革试验区。

（五）治环境，加快美丽金华建设。坚持生态优先，倒逼产业转型，继续强力推进城乡环境综合整治。抓治水，让水清起来。实施“全面提水质”十大工程，消除干流劣V类断面，做好水质保持和提升工作，创建省“清三河”达标县4个以上。实施好286个治污水项目，推进河流沿岸排污口整治，加快城乡截污纳管建设、污水处理厂提标改造，推行第三方运营模式。依法整治印染、化工、造纸、电镀等重污染高耗能行业。加大农业面源污染治理力度，严格执行禁养区、限养区、宜养区规定，加快畜牧业转型升级。实施永久性基本农田土壤治理三年行动计划。完善水源涵养生态功能区政策，加强饮用水源保护。加快实施重点供水工程，市第二水厂2016年6月建成使用。加大水库堤防除险加固和中小河流治理力度，做好防汛抗旱工作。推进节水型城市建设。加快城市排水管网建设改造，试点中水回用。治气尘，让天蓝起来。实施燃煤电厂清洁排放技改三年行动计划，力争2017年所有燃煤电厂和热电厂实现清洁排放。加快燃煤锅炉、工业窑炉、水泥企业等清洁排放技术改造。落实高污染禁燃区制度，关停搬迁大气重污染企业，全面治理餐饮油烟、干洗业废气。市区二环路以内除除夕、正月初一和十五外，禁止燃放烟花爆竹，2016年实现全面禁燃。加大新能源汽车推广力度。实现建筑工地、矿山扬尘控制措施全覆盖，提高城市道路机械化清扫率，市区主要道路实现24小时保洁。加强对雾霾天气的监测预警。治拥堵，让出行畅起来。扎实开展交通治堵“五大行动”， 市县联动治理超限超载。改造优化路网节点，打通断头路。坚持公交优先发展，加快实施BRT（快速公交）项目，加强公共自行车、公共停车场运营管理，倡导文明出行、绿色出行。抓拆改，让城乡美起来。深入开展“无违建”创建活动，力争新创建“无违建县”1个、“无违建乡镇（街道）”20个。大力推进二七区块、石门农场棚户区改造工程，加大背街小巷改善力度，新启动37个城中村改造，其中市区14个。深入开展乱搭乱建、农村非法“一户多宅”等专项整治，完成高铁、高速公路、国道沿线环境整治，拆除违法建筑300万平方米以上，改造城中村210万平方米、旧住宅区120万平方米、旧厂区75万平方米。

（六）建精品，提升新型城镇化水平。认真落实市人大常委会关于深入推进新型城镇化的决议，高标准推进城市规划、建设和管理工作，加快建设精品城市。强化规划引领。按照金华-义乌都市区规划纲要和建设电商新城、丝路新起点、先进装备制造基地、休闲旅游度假基地的目标定位，优化“一轴两带”基础设施空间布局、城镇布局、产业布局，逐步健全中心城市、卫星城市、中心镇和特色镇、中心村和特色村四级规划体系，进一步完善中央商务区、新城区、产业集聚区、生态涵养区等主体功能区规划，编制城市群重大基础设施和配套设施专项规划，强化城市设计，推进“多规合一”。建设中心城区。加快多湖中央商务区开发，构建“三纵四横”道路框架，推进浙中总部经济中心、地区金融服务中心、城市商务创意中心、金华科技文化中心和都市生态游憩中心建设，做美做靓三江六岸景观。加快推进湖海塘、尖峰山、古子城、石门农场等功能区规划建设。推进婺城新城区、金东新城区、金华开发区金西区块建设，完善功能配套，加快产业集聚，提升人气商气。加快金义都市新区建设，重点培育电子商务、现代物流、科技研发和文化创意产业，办好网络经济学院，促进产城融合发展。高标准规划建设义乌丝路新区、陆港新区、科创新区。深化智慧城市、“光网城市”和“宽带中国”示范城市建设。提升城市地下空间开发利用水平。放大同城效应。深入实施都市区建设三年行动计划，统筹推进交通、供水、供电、供气、环保、旅游、通讯等基础设施共建共享、互联互通。加快金温铁路扩能改造工程建设，开工建设金台铁路，加强甬金铁路、金建铁路、金义东城际轨道交通项目前期工作。加快铁路金华站南站房、货场搬迁项目建设，规划建设浙中公铁联运港。统筹推进杭金衢高速改扩建、临金高速、杭绍金台高速、330国道改建、汤九线、金义中线、金兰中线、金义永公路、351国道东磐段等项目建设。加快衢江航运开发。实施永康等输变电工程。加强城市治理。巩固国家卫生城市创建成果，以创建全国文明城市、国家森林城市为载体，以洁化、绿化、美化、亮化、序化、文化为主要内容，加快建设精致集约有内涵的城市。加强市容市貌、市政设施等标准化、精细化管理，完善网格化管理体系。挖掘历史人文资源，打好名人文化牌。

　（七）强“三农”，促进城乡统筹发展。全面加强农业、农村、农民工作，积极推进城乡一体化。加快转变农业发展方式。加强粮食生产功能区和现代农业园区建设，加快农业科技创新，推动多种形式的农村土地流转，鼓励工商资本投资发展设施农业，加快发展绿色生态农业。大力发展旱粮生产。认真抓好“米袋子”“菜篮子”工程。推行农牧结合生态养殖模式，在宜养区扶持生态规模养殖场建设。制订火腿、酥饼、宣莲等传统产品质量标准体系，打响金华有机农业、精品农业、特色农业品牌。扶持发展家庭农场等新型农业主体。培育特色小镇和魅力乡村。统一规划布局，分批分类实施，突出生态休闲、文化体验、新兴产业、传统经典产业等特色，启动婺城环保小镇、磐安江南药镇等10个特色小镇建设。加快小城市培育和中心镇发展，推进城镇基础设施、基本公共服务向农村延伸，有序推进愿意进城的农民转化为城镇居民。深入推进美丽乡村建设，继续实施“12510”精品培育计划，启动“浙中民居”样板建设试点和9条风景线、45个精品村、90个秀美村创建。加大古村落、古民居、古建筑及非物质文化遗产的保护力度，推进20个有故事的魅力乡村建设。促进农民持续增收。加强农民职业技能培训，提升发展来料加工业，支持农民创业基地建设。大力发展农村电子商务，加快建设农产品网络销售平台。加强山区经济发展和精准扶贫工作。支持磐安生态功能区建设，促进金磐扶贫开发区提升发展，加快改善武义柳城、兰溪水亭等民族乡镇生产生活条件。

（八）惠民生，持续增进人民福祉。坚持把惠民利民作为改革发展的出发点和落脚点，实现发展成果更多更公平地惠及全体市民。加强就业社保工作。完善促进就业、鼓励创业、自主择业的政策体系和服务机制，做好高校毕业生等各类群体就业。健全企业工资集体协商制度和最低工资标准调整机制，适时提高基本养老保险待遇标准和城乡居民低保标准，促进城乡居民普遍增收。实施全民参保登记，完善“五费合征”政策，实现应保尽保。推进城乡居家养老照料中心全覆盖，探索“家院互融”新模式，提高养老保障水平。完善社会救助体系，保障困难群众基本生活。推进社会事业改革发展。深化教育改革，提升办学理念，加强师资建设，塑造校园文化，加快推进教育现代化。实施第二轮学前教育三年行动计划。强化学校规划、设计、建设和管理，加大标准化中小学校建设力度，高标准推进义务教育均衡化。提升高中教育质量。推进中职教育特色化发展。实施特殊教育提升计划。支持浙师大、上海财大浙江学院等在金高校发展，办好金华职业技术学院、义乌工商学院等。扶持和规范发展民办教育。深化公立医院改革和基层医疗卫生绩效改革，推行分级诊疗和责任医生签约制度，推进市县中心医院与乡镇社区医院建立双向转诊、资源共享、优势互补的紧密型医联体。加强疾病防控基础建设。加强出生人口性别比综合治理，提升流动人口计生服务水平。大力实施文化惠民工程，推动非遗保护和农村文化礼堂建设，举办施光南音乐节，承办“三月三”畲族文化节，启动编撰金华历史文化系列丛书。开展群众性体育活动，发展体育事业。深入实施妇女儿童发展规划。加快发展老龄事业。加强灾害预警和应急管理工作。创新基层社会治理。推进“信义金华”建设，倡导契约精神，弘扬公序良俗，构建以企业信用为重点的社会诚信体系。深化8890“市域一张网”建设，整合社会资源，完善系统功能，提高专业化、市场化、社会化服务水平。强化普法宣传教育，增强全民法治观念。深化平安金华建设，完善立体化社会治安防控体系，健全矛盾纠纷预防化解机制。加强社区工作者队伍建设，改善工作条件，提升社区公共服务水平。规范发展社会组织，发挥村规民约等积极作用，提升基层自治水平。做好安全生产工作，落实企业主体责任，推进安全生产标准化。加强食品药品安全监管，重视网络安全等工作。办好十方面实事。加大财政投入，切实办好治污清水、气尘合治、公交优先、教育提升、救助保障、扩大就业、食品药品安全、老有所养、文化惠民、城乡公共服务水平提升等十方面民生实事。同时，进一步做好民族、宗教、外侨、对台、民防、气象、统计、档案、地方志、文物保护以及关心下一代等工作。争创全国双拥模范城三连冠。做好援疆、援藏、援青及“双对口”工作。

　　三、加快建设法治政府

　　认真贯彻落实十八届四中全会精神，加快建设职能科学、权责法定、执法严明、公开公正、廉洁高效、守法诚信的法治政府。

加快转变政府职能。坚持法定职责必须为、法无授权不可为，强化依法履职意识，推进政府机构、职能、权限、程序、责任法定化。建立健全“四张清单”动态调整机制，拓展政务服务网功能。深化商事制度和行政审批制度改革，清理审批前置条件，试行“一口式”受理服务，实施“三证合一”登记制。培育和规范中介机构，促进中介服务环节减少、时间压缩、费用节省。全面实行负面清单外企业投资“零地”项目不再审批制度，企业独立选址项目高效审批，非独立选址项目建立市场化要素供给机制。建立健全事中事后监管制度。深化政府购买公共服务改革，落实政府购买法律服务制度，加大政府网上采购力度。依法完善招投标制度，构筑企业法人、中介机构诚信体系，实施“红名单”“黑名单”制度，建立守法诚信褒奖机制和违法失信惩戒机制。

健全依法决策机制。建立市政府常务会议学法制度，完善领导干部任前法律考试制度，开展公务人员法律知识更新培训，提升依法决策、依法行政、依法治理的能力和水平。严格执行重大行政决策公众参与、专家论证、风险评估、合法性审查、集体讨论等法定程序。重大决策出台前向人大报告、与政协协商，建立健全重大决策人大代表、政协委员参与机制。加强规范性文件的合法性审查和备案工作，强化对公共资源利用合同的合法性审查及备案，探索建立重大决策跟踪评估机制。大力推行政府法律顾问制度，加强市、县、乡三级政府法律顾问队伍建设。

规范行政执法行为。推进行政执法体制改革，科学划分执法权限。推进综合执法，减少执法队伍种类，开展跨部门综合执法试点，加快推进乡镇综合执法工作，完善开发区、风景旅游区、水源保护区综合执法体制。落实行政执法责任制，规范管理辅助执法人员。加大对环境保护、城市管理、“五水共治”、“三改一拆”等重点领域执法力度，依法严惩违法行为。落实行政执法案件主办人制度、案件审核制度和重大执法决定法制审核制度。严格执行行政执法自由裁量基准制度。

加大政务公开力度。坚持以公开为常态、不公开为例外原则，推进决策公开、执行公开、管理公开、服务公开、结果公开。向社会全面公开政府职能、法律依据、实施主体、职责权限、管理流程、监督方式等事项。公开行政审批全流程以及行政处罚案件的类别、处罚依据、处罚结果。进一步推进行政权力运行、财政预决算、公共资源配置、公共服务、公共监管、重大项目建设批准和实施、社会公益事业建设等重点领域信息公开。做好政府信息依申请公开工作。

加强作风建设和廉政建设。巩固扩大党的群众路线教育实践活动成果，继续认真落实中央八项规定，严格控制公务消费，深化公务用车改革，坚决遏制“四风”反弹回潮。扎实开展“三严三实”专项教育，强化担当意识和创新精神，全面落实工作责任制和责任追究制，切实增强政府执行力。健全廉政风险防控机制，强化执法监察、廉政监察和效能监察，加强审计监督，重点做好公共资金、国有资产、国有资源和领导干部履行经济责任情况审计工作，把权力关进制度的笼子里，做到干净干事。

各位代表，主动接受监督是建设法治政府的重要内容。市政府将坚决贯彻执行人大及其常委会的决议决定，及时主动向人大及其常委会报告工作。主动接受政协民主监督，自觉接受司法监督、舆论监督和公众监督，广泛听取民主党派、工商联、无党派人士、人民团体和各界人士的意见建议。认真办理提案议案和意见建议，加大领导领衔办理、直接办理力度，改进办理方式，提高办理质量，让广大人民群众更加满意。

各位代表，新的一年，我们站在了新的起点上，责任重大、使命光荣。让我们紧密团结在以习近平同志为总书记的党中央周围，在省委、省政府和市委的坚强领导下，紧紧依靠全市人民，牢牢把握新常态新趋势新机遇，齐心协力，开拓进取，为干好“一三五”、实现“四翻番”，加快建设“两富”“两美”现代化金华而努力奋斗！
